# Causes and Three-year Incidence of Irreversible Visual Impairment in Jing-An District, Shanghai, China from 2010-2015

**DOI:** 10.1186/s12886-017-0603-3

**Published:** 2017-11-28

**Authors:** Fei Xia, Liangcheng Wu, Chenghai Weng, Xingtao Zhou

**Affiliations:** 1 Department of Ophthalmology, Jing-An District Center Hospital of Shanghai (Huashan Hospital Fudan University Jing-An Branch), Shanghai Medical College, Fudan University, Shanghai, China; 2 Department of Ophthalmology & Visual Science, Eye & ENT Hospital, Shanghai Medical College, Fudan University, Shanghai, China

**Keywords:** Blindness, Low vision, Myopia macular degeneration, Diabetic retinopathy

## Abstract

**Background:**

The registry system can be used to observe the distribution trend of diseases and analyze the related data to provide useful information in a way that enables the government to take appropriate interventional measures. The purpose of this study was to determine the causes and three-year incidence of newly registered disabled patients who were blind or had low vision in Jing-An District, Shanghai, China from 2010 to 2015.

**Methods:**

Data from the registration system of visual disability in Jing-An District, Shanghai from 2010 to 2015 were collected and analyzed. In this registry, the only person with permanent visual impairment (VI) was identified as being a certified visually impaired person. The main causes of visual disability were obtained, the three-year incidence of visual disability was calculated, and the relationships between blindness or low vision and age, as well as those between blindness or low vision and gender, were analyzed.

**Results:**

Six-hundred and forty-six newly certified people with VI were registered, including 206 blind patients and 440 low vision patients. The major causes of blindness were myopia macular degeneration (MMD, 23.30%), glaucoma (20.39%), and age-related macular degeneration (AMD, 17.96%). The three leading causes of low vision were MMD (58.86%), AMD (16.36%), and diabetic retinopathy (DR, 7.27%). DR (16.0%) was the first leading cause of blindness and the second leading cause of VI in patients aged 30–59 yrs. from 2010 to 2015. The three-year incidences of blindness in 2010–2012 and 2013–2015(*P* = 0.43), which remained stable throughout this time period, were 32.74/100000 and 36.51/100000, respectively. However, the three-year incidence of low vision was 64.51/100000 in 2010–2012 and 83.58/100000 in 2013–2015(*P* = 0.007), which shows that the incidence increased significantly due to the increase of patients with low vision caused by MMD and DR (*P* = 0.003 and *P* = 0.01, respectively).

**Conclusions:**

MMD, glaucoma, and AMD were the main causes of blindness, while DR was becoming a major cause of VI, especially in working-age people of Jing-An District, Shanghai, China.

## Background

Vision impairments are major global public health problems. In 2002, The World Health Organization (WHO) estimated that 1% of the total global burden of disease, measured as disability-adjusted life years (DALY), was attributable to vision loss. The global burden of disease in 2010 also demonstrated that blindness and visual impairment (VI) were significant public health burdens [1–3]. Estimating these trends is important for several reasons, including for understanding the unmet need and effects of interventions. Registry data can provide valuable information on the characteristics of the relevant population, as well as on details of the services provided. In many cases, these data are annually evaluated and reported, providing an updated source of information on the trends of the incidences and causes of the conditions in question [4, 5]. Jing-An District is one of the nine downtown districts of Shanghai, and it consists of 5 blocks and approximately 300,000 residents. Any citizen of this area with VI can apply for identification to the local Disabled Person’s Federation (DPF), and the Jing-An District center hospital is the only designated work unit for the identification visual disability in this region. Although registration with the DPF is entirely voluntary, it entitles the registered individuals to corresponding practical and monetary benefits. The registration system was described in our previous reports [6, 7]. In this study, we updated the most recent 6 years of data from the registry system with specific objectives, which include the following: (1) evaluating the causal trend of newly registered blind and low vision patients, (2) calculating and comparing the three-year incidences of VI, and (3) suggesting priorities for research and intervention strategies for VI.

## Methods

The registry data for visual disability in the Jing-An District, Shanghai from 2010 to 2015 were collected and analyzed. The study was performed in accordance with the principles of the Declaration of Helsinki for research involving human subjects. The study met all of the standards for ethical approval in China, and the protocol was approved by the municipal government of Shanghai, China.

The criteria for registering blindness and low vision in this study were in accordance with the WHO categories of VI. Blindness was defined as a best spectacle corrected visual acuity (BSCVA) of less than 20/400 (3/60) in the better eye or a corresponding visual field loss to less than 10 degrees in the better eye with the best possible correction. Low vision was defined as a BSCVA of less than 20/60 (6/18) but more than 20/400(3/60) in the better eye. VI was defined as a BSCVA of less than 20/60 (6/18) in the better eye.

All of the registered cases of visual disability needed to undergo an assessment of VI at the Jing-An District center hospital. A comprehensive ocular examination was performed on every applicant, which included uncorrected visual acuity (UCVA), BSCVA, refraction, optical coherence tomography, and color fundus photography. BSCVA was tested using a phoropter (C-200, WEIZHEN Optic Tech. Co., Ltd., China) with a tumbling E letters chart projector at a distance of 2.5 m. Visual field tests were performed using a kinetic arctic perimeter (YZ22, 66 Vision Tech. Co., Ltd., China) when the visual disability was due to glaucoma, retinitis pigmentosa (RP) and other optic nerve diseases.

The causes of blindness were classified according to the International Classification of Diseases, 10th edition [8]. The diagnoses of myopia macular degeneration (MMD), age-related macular degeneration(AMD), glaucoma, RP, corneal opacity, diabetic retinopathy(DR) and other ocular diseases had been previously described [6, 7]. MMD was only considered in subjects with a refractive error exceeding −6.0 diopters in either eye and with one or more of the following ophthalmologic findings: tessellated fundus with yellowish white diffuse or grayish white patchy chorioretinal atrophy, macular hemorrhage or posterior staphyloma. Late AMD was defined by the appearance of either exudative macular degeneration or pure geographic atrophy. Glaucoma was defined according to the International Society for Geographical and Epidemiological Ophthalmology classifications. The diagnoses of RP was based on night blindness, progressive loss of the peripheral visual field, and decreased visual acuity with age, as well as on typical signs observed under fundus examination. The diagnoses of DR, corneal opacity, and other diseases as causes of blindness followed the ophthalmology practice guidelines edited by the China Academy of Ophthalmology.

Using the best judgment, the ophthalmologist attempted to identify the disorder causing the greatest limitation of vision as the cause of blindness or low vision. The causes of blindness or low vision in the better eye were recorded. When two or more causes appeared to have contributed equally to VI for one eye, the primary cause was assigned as the cause. Some treatable eye disorders, such as cataract and refractive error, were not considered as causes of VI in the study. If cataract was regarded as the main cause of VI, the patient was referred for surgery and reassessed at least 2 months postoperatively if their visual function was restored unsatisfactorily.

The statistical analysis was performed using SPSS software version 13 (SPSS, Inc., Chicago, IL). The age-adjusted three-year incidences and the respective 95% confidence intervals (CIs) of blindness and low vision were calculated using the number of cases of VI and the population of Jing-An District in 2010–2012 and 2013–2015 using Microsoft Excel. Age was divided into three age range groups: 1–29 yrs., 30–59 yrs. and 60 yrs. and older. Binary logistic regression was used to analyze the factors related to the occurrences of certified blindness and low vision. Odds ratios (OR) and 95% CIs were determined to describe the influence of age and gender on the incidence of VI. A Chi-squared test was used to analyze differences between the genders and to test the differences in the three-year incidence of VI caused by various pathologies between 2010 and 2012 and 2013–2015. A *P* < 0.05 was considered as being statistically significant.

## Results

### Causes of VI

**Fig.1:**
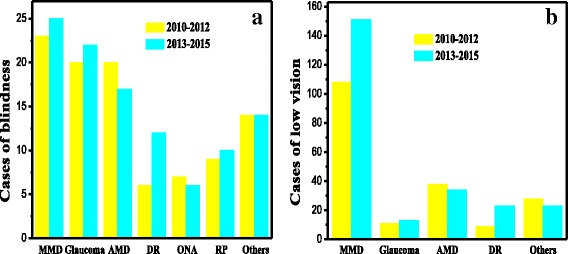
**a** The distribution of the causes of blindness, and **b** the distribution of the causes of low vision in Jing-An District, Shanghai, China from 2010 to 2012 and 2013–2015. Abbreviations: MMD: myopic macular degeneration; AMD: age-related macular degeneration; DR: diabetic retinopathy; ONA: optic nerve atrophy; RP: retinitis pigmentosa

A total of 646 newly certified people with VI were registered as being blind (206 patients) or as having low vision (440 patients) among the approximately 300,000 residents in Jing-An District, Shanghai, China from 2010 to 2015. The main causes of blindness were MMD (23.30%), glaucoma (20.39%), and AMD (17.96%). And 73.0% AMD leading to blindness was wet (neovauscluar), 27.0% was dry; however, only 15.2% AMD leading to low vision was wet, 84.8% was dry. Fig. 1 presents the distribution of the causes of blindness and low vision in Jing-An District, Shanghai, China between 2010 and 2012 and 2013–2015. It can be seen in Fig. 1a that DR was the sixth leading cause of blindness in 2010–2012 and became the fourth leading cause in 2013–2015. According to Fig. 1b, the leading causes of low vision were MMD, AMD and DR.

### Principal causes of VI at different ages

The distribution of the principal causes of VI between the 30–59 yrs. and ≥60 yrs. age groups in Jing-An District from 2010 to 2015 is summarized in Table 1.

**Table 1:** The distribution of the principal causes of VI between the 30–59 yrs. and ≥60 yrs. age groups in Jing-An District from 2010 to 2015

		30–59 yrs. old			≥60 yrs. old		
	Causes	2010–2012 n (%)	2013–2015 n (%)	Total n (%)	2010–2012 n(%)	2013–2015 n (%)	Total n (%)
Blindness	MMD	9(34.6)	7(25.9)	16(30.2)	14(19.2)	18(22.8)	32(21.1)
	Glaucoma	5(19.2)	5(18.5)	10(18.9)	15(20.5)	17(21.5)	32(21.1)
	AMD	0(0)	0(0)	0(0)	20(27.4)	17(21.5)	37(24.3)
	DR	3(11.5)	7(25.9)	10(18.9)	3(4.1)	5(6.3)	8(5.3)
	ONA	3(11.5)	1(3.7)	4(7.5)	4(5.5)	5(6.3)	9(5.9)
	RP	5(19.5)	6(22.2)	11(20.8)	4(5.5)	4(5.1)	8(5.3)
	Others	1(3.7)	1(3.7)	2(3.8)	13(17.8)	13(16.5)	26(17.1)
	Total	26	27	53	73	79	152
Low Vision	MMD	55(78.6)	57(70.4)	112(74.2)	53(42.7)	95(57.9)	148(51.4)
	Glaucoma	1(1.4)	3(3.7)	4(2.6)	10(8.1)	10(6.1)	20(6.9)
	AMD	3(4.3)	3(3.7)	6(3.9)	35(28.2)	31(18.9)	66(22.9)
	DR	1(1.4)	13(16.0)	14(9.3)	8(6.5)	10(6.1)	18(6.3)
	Others	10(14.2)	5(6.2)	15(9.9)	18(14.5)	18(11.0)	36(12.5)
	Total	70	81	151	124	164	288

*Abbreviations: MMD* myopic macular degeneration, *AMD* age-related macular degeneration, *DR* diabetic retinopathy, *ONA* optic nerve atrophy, *RP* retinitis pigmentosa

Two newly registered cases of patients younger than 29 years are not shown in Table 1, including 1 case of blindness caused by retinopathy of prematurity and 1 case of low vision caused by congenital cataract (both of these patients were male). MMD (30.2%), RP (20.8%), DR (18.9%) and glaucoma (18.9%) were the four leading causes of blindness in the patients in the 30–59 yrs. age group, whereas AMD (24.3%), MMD (21.1%) and glaucoma (21.1%) were the three leading causes of blindness in the patients in the ≥60 yrs. age group. Interestingly, DR (25.9%) and MMD (25.9%) equally contributed as the first leading causes of blindness in the patients in the 30–59 yrs. age group in 2013–2015. DR was also the second leading cause of low vision in the patients in the 30–59 yrs. age group in 2010–2015.

### Three-year incidence of VI and age-adjusted three-year incidence of VI

Table 2 shows the three-year incidence of VI attributed to various diseases in Jing-An District, Shanghai from 2010 to 2015.

**Table 2:** Summary of the three-year incidence of VI in Jing-An District, Shanghai from 2010 to 2015

		2010–2012		2013–2015		
		(population: 30.23*10^5^)		(population: 29.31*10^5^)		
	Causes	n	Incidence 1/100000(95%CI)	n	Incidence 1/100000(95%CI)	P
Blindness	MMD	23	7.61(4.50–10.72)	25	8.53(5.19–11.87)	0.69
	glaucoma	20	6.62(3.72–9.51)	22	7.51(4.37–10.64)	0.68
	AMD	20	6.62(3.72–9.51)	17	5.80 (3.04–8.56)	0.69
	DR	6	1.98 (0.40–3.57)	12	4.09 (1.78–6.41)	0.14
	ONA	7	2.31 (0.60–4.03)	6	2.05 (0.41–3.69)	0.83
	RP	9	2.98 (1.03–4.92)	10	3.41 (1.30–5.53)	0.77
	others	14	4.63 (2.21–7.06)	15	5.12(2.53–7.71)	0.79
	Total	99	32.74(26.30–39.20)	107	36.51(29.59–43.42)	0.43
Low vision	MMD	108	35.73(28.99–42.46)	151	51.51(43.30–59.73)	0.003
	AMD	38	12.57(8.57–16.56)	34	11.60(7.70–15.50)	0.73
	Glaucoma	11	3.64(1.49–5.79)	13	4.44(2.02–6.85)	0.63
	DR	9	2.98(1.03–4.92)	23	7.85(4.64–11.05)	0.01
	Others	29	9.59(6.10–13.08)	24	8.19(4.91–11.46)	0.57
	Total	195	64.51(55.45–73.56)	245	83.58(73.13–94.05)	0.007

*Abbreviation: MMD* myopic macular degeneration, *AMD* age-related macular degeneration, *DR* diabetic retinopathy, *ONA* optic nerve atrophy, *RP* retinitis pigmentosa

The three-year incidence of blindness in 2010–2012 was relatively stable with rate of 32.74/100000 in 2010–2012 to 36.51/100000 in 2013–2015 (*P* = 0.43), and that of each cause of blindness remained stable as well (Table 2). However, the increase in the three-year incidence of low vision in 2013–2015 was significant compared with that in 2010–2012 (from 64.51/100000 to 83.58/100000, *P* = 0.007), which was primarily due to the increase in the number of patients with MMD and DR (*P* = 0.003 and *P* = 0.01, respectively).Table 3 summarizes the age-adjusted incidences of blindness and low vision. The age-adjusted three-year incidence of low vision in 2013–2015 was significantly higher than that in 2010–2012(81.12/100000 vs. 66.68/100000, *P* = 0.004). However, there was no difference between the two time periods for any of the age groups (Table 3).

**Table 3:** Summary of the age-adjusted three-year incidence of VI in Jing-An District, Shanghai, China from 2010 to 2015

		2010–2012			2013–2015			
	Age	Population	n	Incidence	Population	n	Incidence	P
	yrs	(10000)		1/100000 (95% CI)	(10000)		1/100000 (95% CI)	
blindness	0–29	7.69	0	0	7.36	1	1.36 (0–4.02)	0.49
	30–59	14.08	26	18.47 (14.84–22.09)	12.47	27	21.65 (13.49–29.82)	0.56
	≥60	8.46	73	86.29 (76.19–96.38)	9.48	79	83.33 (64.96–101.70)	0.83
	Age-adjusted	59.54	204	34.26(29.56–38.96)	59.54	209	35.1(30.34–39.86)	0.81
Low vision	0–29	7.69	1	1.30 (0–3.85)	7.36	1	1.36 (0–4.02)	1
	30–59	14.08	70	49.72(38.07–61.36)	12.47	81	64.96 (50.81–79.10)	0.1
	≥60	8.46	124	146.57(120.79–172.35)	9.48	163	171.94(145.57–198.31)	0.18
	Age-adjusted	59.54	397	66.68(60.12–73.23)	59.54	483	81.12(73.89–88.35)	0.004

### Association of sex and age with VI

The association of sex and age with blindness or low vision was calculated using a logistic regression model, as illustrated in Table 4. The study observed no correlation between blindness and gender (OR = 1.26, *P* = 0.09). However, patients with low vision were more often female (OR = 1.29, *P* = 0.009). The overall number of visually impaired persons was significantly higher in the age ≥ 60 years age group than in the 30–59 years age group (OR = 3.7, *P* < 0.001 for blindness; OR = 2.64, *P* < 0.001 for low vision).

**Table 4:** The association of VI with gender and age in Jing-An District, Shanghai, China from 2010 to 2015

		Blindness			Low vision		
		OR	95%CI	P	OR	95%CI	P
Gender	Male	1			1		
	Female	1.26	0.95–1.66	0.09	1.29	1.07–1.56	0.009
Age	30–59	1			1		
	≥60	3.7	2.70–5.06	<0.001	2.64	2.17–3.21	<0.001

*CI* confidence interval, *OR* odds ratio (binary logistic regression)

## Discussion

The registry system, as one method for disease surveillance, is used to continuously collect the data of diseases, observe the distribution trend of diseases and analyze the relevant information to provide useful guidance for the government to take appropriate interventional measures. Registry data are an important resource for epidemiological studies, especially for those of some rare diseases. The problems related to aging have become increasingly prominent in Shanghai. Due to urban renewal, a large number of residents have moved out of Jing-An District in the past years, leading to a decline in the population. Table 3 shows the trend of the population change in Jing-An District. It is necessary to collect registry data to evaluate the causal trend of blindness and low vision. The present study clearly showed that DR was becoming a major cause of VI, especially in the working age population of Jing-An District, Shanghai, China. As far as we know, this study is the first to assess this topic in China. We observed that 11.2% of blindness was caused by DR and that DR was the fourth leading cause of blindness in 2013–2015. We also observed that 25.9% of blindness was caused by DR (first cause) in patients of the 30–59 yrs. age group in 2013–2015. DR was the second leading cause of low vision in patients of the 30–59 yrs. age group. The three-year incidence of low vision caused by DR was also markedly increased in 2013–2015 compared to that in 2010–2012(*P* = 0.01). DR is the most frequent cause of blindness in those of a working age in developed Western countries [9]. Diabetes is also a major public health problem in China [10]. A recent national survey in 2010 reported that the overall prevalence of diabetes in China was estimated to be 11.6% [11] and that the prevalence of diabetes in Shanghai was 15.91% [12]. A recent meta-analysis including 19 studies performed in mainland China indicated that the prevalence of DR was 23% in the diabetic population [13]. The proportion of blindness caused by DR ranged from 3% to 7% in Southeast Asia and in the Western Pacific region and was as high as 15–17% in developed regions, such as North America and Europe [14]. With the build-up of pressure on the high prevalence of diabetes in China, the incidence of blindness attributable to DR may suddenly and prominently increase in the near future. Our previous study showed that the proportion of blindness caused by DR was only 7.6–8.0% and that DR was fifth or sixth leading cause of blindness in 2001–2009 [6]. However, the proportion of blindness caused by DR was as high as 11.2% in 2013–2015 in the present study. Therefore, we can speculate that DR has become or will become the major cause of VI in the working age population of China in the near future.

It was observed in the present study that 23.30% of blindness cases and 58.89% of low vision cases were due to MMD, which was the first leading cause of VI. This result was consistent with our previous reports [6, 7]. However, this conclusion was different from those of other studies in China. Many reasons can explain this difference, such as socioeconomic development levels, study methods, and the standards of visual impairment. Some treatable diseases, such as cataracts, refractive error and posterior capsule opacity, were extirpated as causes of visual impairment in their studies, and high myopia has been a major cause of blindness and low vision. Tang et al.reported that, in Taizhou, China, 51.1% of low vision cases and 33.4% of blindness cases were caused by MMD if the cataract was extirpated as the cause of VI [15]. Wang et al. reported that MMD was the first leading cause of permanent visual impairment (17.6%) in southern China [16]. Hu et al. also reported that MMD was the leading cause of permanent visual impairment in an aging Chinese metropolitan population (45.9% of the cases were low vision and 42.0% of the cases were blindness) [17]. Many studies also showed that high myopia was emerging as the main cause of blindness in some Asian countries, especially in China [6, 7, 18–20]. High myopia increases the risk for pathologic ocular changes, such as cataract, glaucoma, retinal detachment, and MMD, all of which can cause irreversible vision loss. MMD has been observed to be the most frequent cause of irreversible blindness in China and some other Asian countries.

The present study showed that MMD, glaucoma and AMD were three leading causes of blindness, and that DR was a major cause leading to VI in patients aged 30–59 yrs. Prevention and treatment of these diseases should be one of the key tasks in public health. However, for most people with AMD, vision loss can neither be prevented nor adequately reversed. There is a clear need for further research on the causes and risk factors for AMD. Most patients with glaucoma can maintain sufficient visual acuity if effective treatment is given at an early stage. Therefore, we suggest that local health bureaus should conduct glaucoma screening to diagnose it at an early stage and carry out corresponding chronic disease management strategies in community health centers [21]. MMD is the chief cause of both blindness and low vision. Controlling the development of high myopia is of impending importance. It is necessary to take various measures to control myopia, including pharmacological, environmental, and optical interventions. Studies on the mechanisms of myopia progression are essential for decreasing the vision impairment burden of students. Fortunately, the construction of refractive data for all students has been the focus of school public health officials in Shanghai since 2013 [22]. We hope that this program can help control myopia. DR is also a major cause of VI. The early detection of DR is the key to avoiding blindness. In 2017, screening for DR and remote medical consultation using fundus image data transmission became part of the public health concern in Shanghai. We hope that this project will lead to early detection and active intervention of DR, thus reducing the incidence of VI attributable to DR.

It should be noted that our analysis was based on the definition of legal blindness, which was principally based on BSCVA and visual field data. Meanwhile, our findings did not take into account the incidences of impaired vision and blindness resulting from uncorrected refractive error and cataract, which are potentially important causes of VI that can be reversed by treatment. An inherent limitation of this study has to do with the reliability of reports from registries, which use aggregated information with rigid reporting categories, restricting the capacity for an in-depth analysis. In addition, better reporting or methodological differences, such as an increase in screening level awareness, number of screeners, etc., could account for the variation in the incidences of VI, which did not fully reflect the actual situation. Certainly, a small group of visually impaired individuals remains unregistered in Jing-An District [7].

## Conclusions

In summary, this study identified the three-year incidence and causes of VI in Jing-An District, Shanghai, China from 2010 to 2015. It was observed that MMD was the leading cause of VI. In addition, DR is becoming a major cause of VI, especially in working-age people of Jing-An District, Shanghai, China. Presently, more attention should be paid to diabetics and people with high myopia.

## Abbreviations

AMD: Age-related macular degeneration
BSCVA: Best spectacle corrected visual acuity
DPF: Disabled Person’s Federation
DR: Diabetic retinopathy
MMD: Myopic macular degeneration
ONA: Optic nerve atrophy
RP: Retinitis pigmentosa
UCVA: Uncorrected visual acuity
VI: Visual impairment
WHO: The World Health Organization

## References

[1] Bourne R, Price H, Taylor H, Leasher J, Keeffe J, Glanville J, Sieving PC, Khairallah M, Wong TY, Zheng Y (2013). New systematic review methodology for visual impairment and blindness for the 2010 global burden of disease study. Ophthalmic Epidemiol.

[2] Bourne R, Price H, Stevens G (2012). Global burden of visual impairment and blindness. Arch Ophthalmol.

[3] Lim SS, Vos T, Flaxman AD, Danaei G, Shibuya K, Adair-Rohani H, Amann M, Anderson HR, Andrews KG, Aryee M (2012). A comparative risk assessment of burden of disease and injury attributable to 67 risk factors and risk factor clusters in 21 regions, 1990-2010: a systematic analysis for the global burden of disease study 2010. Lancet.

[4] Bener A, Zirie MA, Kim EJ, Al BR, Zaza M, Al-Nufal M, Basha B, Hillhouse EW, Riboli E (2012). Measuring burden of diseases in a rapidly developing economy: state of Qatar. Glob J Health Sci.

[5] Skaat A, Chetrit A, Belkin M, Kinori M, Kalter-Leibovici O (2012). Time trends in the incidence and causes of blindness in Israel. Am J Ophthalmol.

[6] Wu L, Sun X, Zhou X, Weng C (2011). Causes and 3-year-incidence of blindness in Jing-an district, shanghai, China 2001-2009. BMC Ophthalmol.

[7] Wu LC, Sun XH, Zhou XT, Wen CH (2013). Unrecognized and unregistered blindness in people 70 or older in Jing'an district, shanghai, China. Int J Ophthalmol.

[8] DeAlmeida DR, Watzlaf VJ, Anania-Firouzan P, Salguero O, Rubinstein E, Abdelhak M, Parmanto B (2014). Evaluation of inpatient clinical documentation readiness for ICD-10-CM. Perspect Health Inf Manag.

[9] Congdon NG, Friedman DS, Lietman T (2003). Important causes of visual impairment in the world today. JAMA.

[10] He J, Gu D, Wu X, Reynolds K, Duan X, Yao C, Wang J, Chen CS, Chen J, Wildman RP (2005). Major causes of death among men and women in China. N Engl J Med.

[11] Xu Y, Wang L, He J, Bi Y, Li M, Wang T, Wang L, Jiang Y, Dai M, Lu J (2013). Prevalence and control of diabetes in Chinese adults. JAMA.

[12] Qin Y, Wang R, Ma X, Zhao Y, Lu J, Wu C, He J. Prevalence, awareness, treatment and control of diabetes mellitus-a population based study in shanghai, China. Int J Environ Res Public Health. 2016;13 10.3390/ijerph13050512 PMC4881137 27213415

[13] Liu L, Wu X, Liu L, Geng J, Yuan Z, Shan Z, Chen L (2012). Prevalence of diabetic retinopathy in mainland China: a meta-analysis. PLoS One.

[14] Resnikoff S, Pascolini D, Etya'Ale D, Kocur I, Pararajasegaram R, Pokharel GP, Mariotti SP (2004). Global data on visual impairment in the year 2002. Bull World Health Organ.

[15] Tang Y, Wang X, Wang J, Huang W, Gao Y, Luo Y, Lu Y (2015). Prevalence and causes of visual impairment in a Chinese adult population: the Taizhou eye study. Ophthalmology.

[16] Wang L, Huang W, He M, Zheng Y, Huang S, Liu B, Jin L, Congdon NG, He M (2013). Causes and five-year incidence of blindness and visual impairment in urban southern China: the Liwan eye study. Invest Ophthalmol Vis Sci.

[17] JY H, Yan L, Chen YD, XH D, Li TT, Liu DA, DH X, Huang YM, Wu Q (2017). Population-based survey of prevalence, causes, and risk factors for blindness and visual impairment in an aging Chinese metropolitan population. Int J Ophthalmol.

[18] Holden B, Sankaridurg P, Smith E, Aller T, Jong M, He M (2014). Myopia, an underrated global challenge to vision: where the current data takes us on myopia control. Eye (Lond).

[19] Iwase A, Araie M, Tomidokoro A, Yamamoto T, Shimizu H, Kitazawa Y (2006). Prevalence and causes of low vision and blindness in a Japanese adult population: the Tajimi study. Ophthalmology.

[20] Varma R, Kim JS, Burkemper BS, Wen G, Torres M, Hsu C, Choudhury F, Azen SP, McKean-Cowdin R (2016). Prevalence and causes of visual impairment and blindness in Chinese American adults: the Chinese American eye study. JAMA Ophthalmol.

[21] Sun XH (2017). Focus on popular science education of glaucoma and reduce glaucomatous low vision and blindness. Zhonghua Yan Ke Za Zhi.

[22] Chu RY, XM Q (2009). Setting the individual file of ocular refractive development of children is the primary procedure in the prevention of myopia. Zhonghua Yan Ke Za Zhi.

